# Primary care challenges of an obscure case of “Alice in Wonderland” syndrome in a patient with severe malaria in a resource-constrained setting: a case report

**DOI:** 10.1186/s12879-017-2918-3

**Published:** 2017-12-22

**Authors:** Benjamin Momo Kadia, Cyril Jabea Ekabe, Ettamba Agborndip

**Affiliations:** 1 Foumbot District Hospital, Foumbot, Cameroon; 2 Grace Community Health and Development Association, Kumba, Cameroon; 3 Mbonge District Hospital, Mbonge, Cameroon; 4 Health Education and Research Organization, Buea, Cameroon; 5 Ekondo-Titi District Hospital, Ekondo-Titi, Cameroon

**Keywords:** “Alice in Wonderland” syndrome-severe malaria-quinine-case report

## Abstract

**Background:**

**“**Alice in Wonderland” syndrome (AIWS) is a rare neurological abnormality characterized by distortions of visual perceptions, body schema and experience of time. AIWS has been reported in patients with various infections such as infectious mononucleosis, H1N1 influenza, Cytomegalovirus encephalitis, and typhoid encephalopathy. However, AIWS occurring in a patient with severe malaria is less familiar and could pose serious primary care challenges in a low-income context.

**Case presentation:**

A 9-year-old male of black African ethnicity was brought by his parents to our primary care hospital because for 2 days he had been experiencing intermittent sudden perceptions of his parents’ heads and objects around him either “shrinking” or “expanding”. The visual perceptions were usually brief and resolved spontaneously. One week prior to the onset of the visual problem, he had developed an intermittent high grade fever that was associated with other severe constitutional symptoms. Based on the historical and clinical data that were acquired, severe malaria was suspected and this was confirmed by hyperparasitaemia on blood film analysis. The patient was treated with quinine for 10 days. Apart from a single episode of generalized tonic-clonic seizures that was observed on the first day of treatment, the overall clinical progress was good. The visual illusions completely resolved and no further abnormalities were recorded during 3 months of follow-up.

**Conclusion:**

Symptoms of AIWS usually resolve spontaneously or after treatment of an underlying cause. In our case, the successful treatment of severe malaria coincided with a complete regression of AIWS whose aetiology was poorly-elucidated given the resource constraints. In any case, the good outcome of our patient aligns with previous reports on acute AIWS that highlight a limited need for excessive investigation and treatment modalities which are, in passing, predominantly unaffordable in resource-limited primary care settings.

## Background

“Alice in Wonderland” syndrome (AIWS) is a rare and complex neurological disorder characterized by distortions of visual perceptions, body schema and experience of time [1–4]. The disorder has been reported in patients with various infections such as Epstein-Barr virus encephalitis, H1N1 influenza, Cytomegalovirus infection, Coxsackie B1 virus encephalitis, Varicella-Zoster encephalitis, typhoid encephalopathy and scarlet fever [1, 5].

Severe malaria may be a life threatening condition and it is frequently encountered in developing countries [6, 7]. It is primarily linked with *Plasmodium falciparium* malaria. Previous reports suggest that severe malaria could have atypical presentations and could even mimic pathologies such as psychosis and rabies [8]. These atypical manifestations of the disease could lead to diagnostic delay and increase morbidity and mortality. Here, we report an unusual case of symptoms of AIWS in a child with severe malaria and we highlight the associated primary care challenges in a resource-limited context.

## Case presentation

A 9-year-old male of black African ethnicity was brought by his parents to our primary care hospital because for 2 days he had been experiencing intermittent sudden perceptions of his parents’ heads either “shrinking” or “expanding” although he could still recognize their faces. He also had the same perception of objects around him. The visual perceptions were usually brief, resolved spontaneously and did not involve confusion in colours or individuals. For fear of a magico-religious origin of the illness, his parents took him to a traditional healer where he was given various herbal concoctions to drink for 2 days but there was no regression of the symptoms.

It is worth mentioning that 1 week prior to the onset of the visual symptoms the boy had developed an intermittent high grade fever which persisted in spite of the oral antimalarial (arthemeter and lumefantrin combination) and antipyretic medications (paracetamol and ibuprofen) which his parents gave to him at home. The fever was associated with general weakness, loss of appetite and severe polyarthralgia.

The review of systems revealed severe headache and postprandial vomiting. The child did not have abnormal auditory or tactile perceptions. This was his first time of having visual problems. He was diagnosed with and treated for severe malaria at the ages of 2 and 6 but visual disturbances were not reported on these occasions. His past history was otherwise without particularity.

On admission, the boy still experienced the visual distortions. Clinical examination revealed an extremely lethargic patient with pale conjunctivae and icteric sclerae. His Glasgow coma score was 15/15 and he was oriented in person, place and time. His vital signs were: blood pressure: 70/50 mmHg, pulse: 117beats/min, respiratory rate: 29breaths/min, and temperature: 36 °C. There was mucocutaneous pallor. Neurological examination revealed generalized hypotonia but there were neither focal neurological nor meningeal signs. Head and neck examination as well as the rest of the physical examination was without notable findings as well.

Severe malaria was suspected. Typhoid encephalopathy and hepatic encephalopathy were considered as differential diagnoses. A series of laboratory tests were then requested:

1. To confirm the diagnosis of malaria, a thick blood film analysis was done which was remarkable for hyperparasitaemia at 277,000 trophozoites/μl (obtained from the product of the number of parasites counted per 100 white cells on the thick blood smear and the number of white cells per microliter of blood),
2. A full blood count revealed a haemoglobin value of 8.3 g/dl (moderate anaemia) and a platelet count of 101,000 platelets/mm^3^ (moderate thrombocytopenia)
3. The random blood sugar was 50 mg/dL (moderate hypoglycaemia)
4. The Widal test was used to check the possibility of typhoid and the titre was weakly positive.
5. Hepatitis B and C antigens were negative and liver enzymes were within normal limits

In view of prostration and hyperparasitaemia, the boy was diagnosed with severe malaria. The patient was placed on quinine 300 mg in 300 ml of glucose infusion to flow over 4 h and to be repeated 8 hourly. He was also placed on intravenous paracetamol at a dose of 450 mg 6 hourly. Two hours after the first quinine and glucose infusion, he had an episode of generalized tonic-clonic seizures that were arrested with slowly administered intravenous diazepam. Meningo-encephalitis was considered as a possible aetiology of the neurological problems. Subsequent lumbar puncture and cerebrospinal fluid analysis were without particularity. The quinine and glucose infusions were continued for 5 days and the clinical progress was good. Oral relay of the antimalarial drug was done with Quinine tablets at a dose of 900 mg daily in three divided doses for 5 days. The patient was discharged after 8 days of hospitalization and reviewed on out-patient basis. A repeat of the thick blood film analysis 2 weeks after discharge was negative for malaria parasites. The visual symptoms which were experienced by the boy were suggestive of AIWS and they had completely resolved after the successful treatment of malaria. No further visual abnormalities were reported during 3 months of follow-up and reassurance.

## Discussion and conclusions

Malaria is a major global health problem [9]. Severe malaria can be fatal in the absence of early recognition of its complications and/or delay in appropriate treatment. Therefore, prompt action is particularly necessary for vulnerable groups such as young children [10, 11]. We present an atypical case of severe malaria associated with symptoms of AIWS. In spite of the positive outcome, it is worth mentioning that our approach to the case was flawed by adversities which could not be surmounted in our resource-constrained primary care setting. Serological and/or virological tests to rule out known causes of AIWS such as infectious mononucleosis, coxsackie B virus and mycoplasma encephalitis were lacking. The absence of appropriate resources to perform a standard test for typhoid such as stool antigen test or blood culture compelled us to rule out typhoid encephalopathy (which was an important aetiological differential diagnosis of AIWS) with the Widal test which is still widely used in developing countries although it is considered obsolete [12]. Finally, since diagnosing and treating malaria usually takes precedence over establishing the species of malaria parasite especially in falciparium malaria endemic areas, a thin blood film analysis to verify the species of malaria parasite that may have been associated with this atypical manifestation was underestimated.

Whilst infectious diseases, especially Epstein Barr Virus infection, account for a considerable proportion of previously reported cases of AIWS [1], no previous report has described the occurrence of symptoms of AIWS in a patient with severe malaria and there is no known association between the two pathologies. AIWS was first described in 1955 by the British psychiatrist John Todd. The name refers to Lewis Carroll’s renowned children’s book Alice’s Adventures in Wonderland, in which Alice used to feel (among other things) that her body grew both larger and smaller [13]. AIWS is a rare and poorly understood clinical syndrome whose diagnosis requires profound knowledge of its varying symptoms (which include about 42 visual symptoms and 16 non-visual symptoms) as well as proper history-taking and thorough physical examination [1]. In general, the symptoms of AIWS need to be distinguished from other disorders of perception such as hallucinations and illusions, with which they may be easily confused [1]. These make the diagnosis of AIWS challenging, added to the fact that there is lack of a consensus on its diagnostic criteria [5].

It is important to establish the most likely aetiology of AIWS and it should be ascertained whether the suspected aetiology could be responsible for the symptoms of AIWS. Recent studies suggest that AIWS is more frequent in persons less than 18 years of age and most of the patients present with micropsia (seeing things smaller than they are) and/or macropsia (seeing things larger than they are) [1] as in our experience. However, in patients below 18 years of age, encephalitides are the main mediators of the symptoms of AIWS [1] and in our patient, cerebral malaria which was the most relevant encephalopathy to consider, was less likely in the absence of unarousable coma. These notwithstanding, the patient’s historical, clinical and laboratory data associated with the episode of seizures in the course of treatment may be suggestive of a possible cerebral lesion leading to AIWS although it is obscure if this lesion was associated with neurological complications of severe malaria or an undiagnosed co-existing familiar cause of AIWS including Epstein Barr virus, influenza, partial seizures, or encephalomyelitis. The seizures observed in our patient may have also been due to quinine-induced hypoglycaemia. Albeit current literature proposes that hyperpyrexia (which is a common feature of severe malaria) could cause AIWS [1], this was also a less plausible aetiology in our patient whom on admission experienced visual distortions in the absence of fever. Finally, while it is known that some drugs like mefloquine (an antimalarial) and topiramate (an anticonvulsant) could trigger symptoms of AIWS, our patient’s drug history was unrevealing in this regard.

The role of paraclinical investigations in evaluating patients suspected of having symptoms of AIWS remains controversial. Some authors recommend auxiliary investigations including imaging techniques such as magnetic resonance imaging and electroencephalogram, particularly in cases with long-standing or recurring symptoms of AIWS in whom specific neurobiological correlates of the individual symptoms may be identified [1]. Other authors discount the relevance of imaging techniques in the isolation of specific pathological brain lesions which could explain the symptoms of AIWS [14, 15]. In any case, the symptoms of AIWS usually resolve either spontaneously or after adequate therapy directed towards a suspected underlying cause and as such the symptoms of AIWS are generally considered to be benign [1, 16]. In our case, the successful treatment of severe malaria possibly coincided with the characteristic spontaneous resolution of AIWS whose cause was poorly-elucidated.

In conclusion, AIWS is a rare clinical syndrome with a myriad of possible causes. It therefore presents significant diagnostic and therapeutic challenges. Symptoms of AIWS generally resolve spontaneously or after treatment of an underlying aetiology. The regression of AIWS in our patient may have been consequent to the characteristic self-limiting nature of the syndrome which possibly coincided with the successful treatment of severe malaria (which was less likely the cause of AIWS). In any case, in line with previous reports on acute cases of AIWS, the good clinical progress of our patient further suggests a limited need for excessive investigation and treatment modalities which are, by the way, predominantly unaffordable in resource-limited primary care settings.

## Abbreviation

AIWS: “Alice in Wonderland” syndrome
